# Laboratory Markers of Ventricular Arrhythmia Risk in Renal Failure

**DOI:** 10.1155/2014/509204

**Published:** 2014-05-26

**Authors:** Ioana Mozos

**Affiliations:** Department of Functional Sciences, “Victor Babes” University of Medicine and Pharmacy, T. Vladimirescu Street 14, 300173 Timisoara, Romania

## Abstract

Sudden cardiac death continues to be a major public health problem. Ventricular arrhythmia is a main cause of sudden cardiac death. The present review addresses the links between renal function tests, several laboratory markers, and ventricular arrhythmia risk in patients with renal disease, undergoing or not hemodialysis or renal transplant, focusing on recent clinical studies. Therapy of hypokalemia, hypocalcemia, and hypomagnesemia should be an emergency and performed simultaneously under electrocardiographic monitoring in patients with renal failure. Serum phosphates and iron, PTH level, renal function, hemoglobin and hematocrit, pH, inflammatory markers, proteinuria and microalbuminuria, and osmolarity should be monitored, besides standard 12-lead ECG, in order to prevent ventricular arrhythmia and sudden cardiac death.

## 1. Introduction

Cardiovascular diseases continue to be the leading mortality cause worldwide. Cardiac and renal diseases frequently coexist and significantly increase mortality, morbidity, and the complexity and cost of care [1]. Cardiovascular diseases and complications are the major causes of death in patients with chronic kidney disease and on dialysis [2–4]. Impaired renal function is associated with worse clinical outcomes in patients with myocardial infarction, heart failure, and left ventricular systolic dysfunction [5, 6]. Syndromes describing the interaction between heart and kidney have been defined as cardiorenal syndromes to indicate the bidirectional nature of the various syndromes [1]. The incidence of the cardiorenal syndrome has increased due to the enhanced longevity of the population. Patients survive more years with cardiac or renal dysfunction [7].

Sudden cardiac death is an unexpected death from a cardiovascular cause with or without structural heart disease [8]. It is very often caused by ventricular arrhythmia.

The present review will address the links between renal function tests, several laboratory markers, and ventricular arrhythmia risk in patients with renal disease, undergoing or not hemodialysis or renal transplant, focusing on recent clinical studies.

## 2. Electrocardiographic Predictors of Ventricular Arrhythmia and Sudden Cardiac Death

Several electrocardiographic (ECG) methods can be used to assess ventricular arrhythmia risk, including measurement of the QT interval, Tpeak-Tend interval [9], and QT dispersion on the standard 12-lead ECG. The QT interval is the electrocardiographic expression of ventricular depolarization and repolarization and, if prolonged, a predictor of fatal ventricular arrhythmias and sudden cardiac death [10, 11]. QT dispersion, the range of interlead differences of the QT interval, was considered as an index of spatial inhomogeneity of repolarization duration [12]. It can be calculated as the difference between the longest and the shortest QT interval in all measurable leads. Despite simplicity, the measurement methodology and normal values have not been standardized and the sensitivity and specificity of abnormal values were low [13]. No superior alternative to the noninvasive methods has been found; thus, the data on QT dispersion should be further considered [14].

Signal averaged ECG (SA-ECG) is a method used to detect late ventricular potentials (LVPs), averaging approximately 300 ECG cycles, in order to detect late ventricular potentials, by minimizing the noise level [15]. LVPs are low amplitude, high frequency waveforms, appearing in the terminal part of the QRS complex [16]. LVPs are present, if, according to an international convention, at least 2 of the following 3 criteria are positive: SAECG-QRS duration >120 ms, low amplitude signal (LAS40; the duration of the terminal part of the QRS complex with an amplitude below 40 *μ*V) >38 ms, and root mean square signal amplitude of the last 40 ms of the signal (RMS40) <20 *μ*V [17].

Heart rate variability (HRV), recorded by continuous electrocardiography over a 24-hour period, is a noninvasive measure of autonomic dysfunction and a risk factor for cardiovascular disease [18]. Decreased heart rate variability is an independent risk factor for sudden cardiac death [19].

## 3. Renal Function

Recently, even a slight impaired kidney function has been associated with cardiovascular disease [20].

Several observational studies have demonstrated an association between moderate kidney dysfunction and sudden cardiac death in people with cardiovascular disease [21–23]. It was difficult to conclude whether kidney dysfunction is an independent predictor of sudden cardiac death.

Significant correlations and associations were obtained between signal averaged electrocardiography criteria and* serum creatinine* and* estimated glomerular filtration rate* (Modification of Diet in Renal Disease equation, Cockroft-Gault equation, and Salazar-Corcoran equation for obese patients) in hypertensive patients [24]. Despite the high prevalence of signal averaged electrocardiography abnormalities in patients with left ventricular hypertrophy, the later was not a sensitive or specific predictor for late ventricular potentials or abnormal signal averaged ECGs in the study of Mozos et al. [24].

Mild-to-moderate kidney dysfunction, assessed by the estimated glomerular filtration rate, is associated with a significant elevated risk of ventricular fibrillation in acute ST elevation myocardial infarction [25].

Several other new markers of renal function have been described, including neutrophil gelatinase associated lipocalin (NGAL), predicting mortality in heart failure patients, with and without chronic kidney disease [26], and adverse cardiac events in ST segment elevation myocardial infarction patients treated with primary percutaneous coronary intervention [27]. NGAL is a glycoprotein released by the damaged renal tubular cells and a marker of clinical and subclinical acute kidney injury [27] and in-hospital mortality in the emergency department, enabling clinicians to distinguish between chronic and early reversible kidney damage and to identify patients needing renal replacement therapy [28]. No study addressed yet the relation between NGAL and ventricular arrhythmia risk. A link could exist, considering that NGAL is expressed in endothelial cells, smooth muscle cells, and macrophages in atherosclerotic plaques and may be involved in the development of atherosclerosis via endothelial dysfunction, inflammation and matrix degradation, and plaque instability [27], and NGAL is an earlier marker of acute kidney injury than serum creatinine [28]. NGAL could be an important biomarker for mirroring acute cardiac diseases, kidney damage and arrhythmias.

“Culprit” biomarkers including soluble ST2 and Galectin 3, reflecting cardiac and renal fibrosis, could also be linked to an increased arrhythmia risk. Galectins are a family of soluble beta-galactoside-binding lectins that play regulatory roles in inflammation, immunity, and cancer [29]. Galectin 3 was associated with fibrogenesis in the heart, kidney, and liver. A role for Galectin 3 in the pathophysiology of heart failure has been demonstrated, related to its stimulatory effect on macrophage migration, fibroblast proliferation, and the development of fibrosis [29]. In the kidneys, Gal 3 protects renal tubules from chronic injury by limiting apoptosis and it may be an important factor in matrix turnover and fibrosis attenuation [30]. Soluble ST2 is part of the interleukin 1 receptor family, and binding of interleukin 33 to soluble ST2 reduces binding to ST2 receptors, enabling cardiac fibrosis and hypertrophy [31]. Elevated soluble ST2 concentrations were predictive of sudden cardiac death in patients with chronic heart failure and may have an impact on clinical decision-making [32]. ST2 is a biomarker enabling identifying patients with low survival benefit from implantable cardioverter defibrillator therapy [33].

## 4. Renal Disease 

Progressive renal disease is associated, from the earliest stages, with increased QT interval duration and dispersion and with an increased risk of cardiovascular death, especially sudden death. A stepwise increase in mortality for each stage of chronic kidney disease was found [34]. QTc prolongation and torsade de pointes are associated with end stage renal disease and they can cause sudden cardiac death [35].

It has also been reported that left ventricular mass is increased from the earliest stages of renal disease (near normal renal function), linked to increased QT interval and dispersion, and with minor rhythm abnormalities, providing a link with the high risk of sudden death in this population too [35]. Besides left ventricular hypertrophy, systolic and diastolic dysfunction, interstitial fibrosis, and autonomic neuropathy were found among patients with end stage renal disease [36, 37].

The risk of sudden cardiac death is dependent on the severity of chronic kidney disease [3]. A 10 mL/min reduction in* creatinine clearance* was associated with an increased risk of sudden cardiac death in a retrospective study of patients undergoing implantable cardioverter defibrillator (ICD) implantation for primary prevention of sudden cardiac death [38]. Hager et al. compared death at 1 year in 958 patients with chronic kidney disease, who had undergone ICD placement, and concluded that the mortality rate at 1 year increases with worsening chronic kidney disease and if left ventricular dysfunction was present [3].

*Cystatin C*, a cysteine protease inhibitor, produced by all nucleated cells, released into the bloodstream, undergoes glomerular filtration, and metabolization in the proximal tube [39]. It is considered a potential endogenous filtration marker and estimates the glomerular filtration rate better than serum creatinine [39]. Impaired kidney function, assessed by cystatin C, is independently associated with sudden cardiac death risk among elderly persons without clinical cardiovascular disease [40]. Participants meeting the definition of “preclinical kidney disease” (an estimated GFR >60 mL/min per 1.73 m^2^) had also an elevated risk of sudden cardiac death, equivalent to participants with chronic kidney disease [40]. Cystatin C levels and the corresponding cystatin-based eGFR estimates better the risk of sudden cardiac death among elderly persons than creatinine, considering that creatinine is an insensitive measure of kidney function in elderly persons [40, 41]. It should also be mentioned that elevated cystatin C concentrations also capture preclinical kidney disease [40].

Sudden cardiac death may be a direct result of kidney dysfunction. An increased prevalence of left ventricular hypertrophy and systolic and diastolic dysfunction were found among patients with kidney disease, including those with elevated cystatin C concentrations, which could explain the increased risk of sudden cardiac death [37]. Autonomic dysfunction, myocyte dysfunction, altered electrolyte metabolism, and cardiac fibrosis may also contribute to arrhythmic risk in patients with kidney dysfunction [40].

Electrolyte imbalances are common in patients with acute and chronic renal failure, especially hyperkalemia and hypocalcemia. Electrolyte disorders can alter cardiac ionic currents kinetics and can generate or facilitate cardiac arrhythmias [42]. Potassium, calcium, sodium, and magnesium play a role in the genesis of experimental arrhythmias; however, in the clinical setting, only altered potassium concentration is responsible for the majority of arrhythmias [43].

*Hyperkalemia* appears in patients with acute renal failure due to impaired renal excretion associated with oliguria or anuria, transmineralization, or due to cellular damage. It occurs late in chronic kidney disease, at significant reductions of the glomerular filtration rates, but is a common potentially fatal complication [44]. There are several other additional causes of hyperkalemia in patients with chronic kidney disease, including high dietary potassium intake relative to the residual renal function, an extracellular shift of potassium caused by the metabolic acidosis, and therapy with renin-angiotensin-aldosterone system blockers that inhibit renal potassium excretion [45]. Hyperkalemia reduces the resting membrane potential, slows conduction velocity, increases the rate of depolarization and repolarization due to increased membrane permeability for potassium, and shortens action potential duration [42, 45, 46]. Additional electrolyte disturbances in renal patients may influence the cardiac membrane potential [45]. The ECG manifestations of hyperkalemia include tall, “tented,” peaked, narrow-based T waves, decreased amplitude of the R wave, delayed atrioventricular and intraventricular conduction delay with prolonged PR interval and widened QRS complex, blending of the QRS complex into the T wave (the sine wave), ST segment depression, QT interval shortening, decreased amplitude or disappearance of the P wave, accelerated junctional rhythm, ventricular tachycardia and fibrillation, and asystole [42, 43, 47]. Thus, hyperkalemia can induce deadly cardiac arrhythmias [47]. In patients with acutely elevated serum potassium levels due to potassium intoxication or renal failure, a pseudomyocardial infarction pattern may appear: ST segment elevation, secondary to derangements in myocyte repolarization, and lowering of serum potassium level by hemodialysis were associated with return of the electrocardiogram toward normal [48]. The thresholds of serum potassium, above which changes in the ECG are manifest, differ from patient to patient [44]. Patients with chronic kidney disease or chronic hyperkalemia may develop compensatory mechanisms, enabling restoring of the myocardial membrane potential to normal [49], which could explain normal ECGs despite very high serum potassium (>9 mmol/L) [50]. The ECG cannot reliably be used to exclude the presence of hyperkalemia or to monitor therapy designed to reduce serum potassium [50].

Loss of glomerular filtration rate explains why hyperkalemia is one of the most common reasons for emergency dialysis [44]. Green et al. demonstrated in a study of 145 patients with ESRD that hyperkalemia was not significantly predictive of T wave tenting, especially in older patients, considering that T wave amplitude decreases with age, and in diabetic patients [44]. On the other hand, in the absence of a baseline ECG for comparison, one cannot determine whether T wave tenting is due to an associated coronary heart disease, left ventricular hypertrophy, and acidosis or due to hyperkalemia [44, 51]. Dreyfuss et al. reported a positive correlation between the amplitude of the T wave in V2 and the arterial concentration of H^+^ and a negative correlation with the arterial total CO_2_ content [51].

Hyperkalemic events in patients without chronic kidney disease were associated with higher mortality than in patients with chronic kidney disease, due to an adaptive response that leads to a new increased steady state serum potassium level and an increased gut potassium excretion [45]. The reduced sensitivity to cardiac complications due to hyperkalemia in patients with chronic kidney disease is due to chronic hyperkalemia, which is better tolerated. An inverse relationship was found between the severity (stage) of chronic kidney disease and mortality after a hyperkalemic event [45].

*Hypocalcemia*, most frequently seen in chronic renal failure, appears due to hyperphosphatemia and reduced renal hydroxylation of vitamin D, with impaired calcium absorption, and causes secondary hyperparathyroidism. Hypocalcemia results in decreased contractility, increased excitability, and prolonged QT intervals and T wave alterations [42, 52].

Hypocalcemia is usually associated with other electrolyte abnormalities in chronic renal failure. The combination of hyperkalemia and hypocalcemia has a cumulative effect on the atrioventricular and intraventricular conduction and facilitates ventricular fibrillation [42]. A previous study found an inverse relationship between serum calcium and the T wave amplitude, hypothesizing that, in this case, hypercalcemia was cardioprotective from the effects of hyperkalemia and masked the hyperkalemic ECG changes [53].

Voiculescu et al. analyzed 68 patients with chronic renal failure and found a prolonged QT interval in 11.8% of the patients [54]. QT prolongation correlated with the number of years of renal failure, serum concentrations of potassium, and calcium and diastolic blood pressure but was not dependent on the level of serum magnesium, phosphates, hemoglobin or bicarbonate, and the type of renal substitution (hemodialysis or continuous ambulatory peritoneal dialysis) [54]. During the follow up of 3.8 months, no cases of sudden cardiac death and no significant arrhythmia incidence were detected [54].

Magnesium, the second most abundant intracellular cation after potassium, is usually ignored outside critical care. Prevalence of hypomagnesemia in hospitalized patients is approximately 20% [55], and critical serum magnesium level is associated with seizures and life-threatening arrhythmias [56]. In the presence of low calcium concentrations, magnesium deficiency prolongs the action potential plateau [42]. Patients with acute myocardial infarction and hypomagnesemia have higher mortality due to ventricular arrhythmias secondary to a lower threshold for depolarization [57]. Patients with renal failure develop* hypermagnesemia* due to an impaired renal excretion. Low magnesium can appear due to an excessive urinary loss: diuresis due to alcohol; glycosuria in diabetes mellitus and diuretics [56]; therapy with nephrotoxic drugs including cisplatin and amphotericin B; use of bisphosphonates, cyclosporine, malabsorption, or malnutrition; formation of insoluble complexes with phosphorus; shifts from the extracellular to the intracellular fluid (due to acidosis, insulin); transdermal losses [55, 56]. Patients with* hypomagnesemia* had a higher mortality than those with normal levels of magnesium [55]. Considering that magnesium is required for cellular function, its deficiency will probably contribute to organ system failure [55]. Severe hypomagnesemia is often accompanied by hypocalcemia and hypokalemia, and both are refractory to therapy until magnesium has been repleted [55]. The contribution of hypomagnesemia is difficult to ascertain for ventricular arrhythmias, considering the associated electrolyte abnormalities, and clinicians, very often, fail to measure magnesium.

Foglia et al. reported prolonged QT intervals in primary renal hypokalemia-hypomagnesemia, confirming that potassium and magnesium depletion prolong the duration of the action potential of the cardiomyocyte [58]. The altered ventricular repolarization is, especially, linked to their concentration gradient across the cardiomyocyte membrane [58]. On the other hand, continuous ambulatory electrocardiography and exercise testing failed to detect clinically relevant arrhythmias in patients with renal hypokalemia [58–60].

Lower values of heart rate variability were found in patients with chronic renal failure [61]. Lower heart rate variability was significantly associated with older age, female gender, diabetes, higher heart rate, C-reactive protein and phosphorus, lower serum albumin, higher high-density lipoprotein, and stage 5 chronic kidney disease in patients with nondialysis chronic kidney disease [18]. C-reactive protein is a known cardiovascular risk factor, and autonomic function may be impaired due to inflammation [62].

*Hyperphosphatemia*, highly prevalent among patients with end stage renal disease, especially if higher than 6.5 mg/dL, contributes to cardiac mortality, including sudden death [63]. The relative risk of sudden death in patients with hyperphosphatemia was strongly associated with elevated calcium-phosphate product and elevated serum parathyroid hormone levels [63]. It is speculated that elevated phosphates may increase vascular calcification and smooth muscle proliferation and may impair myocardial perfusion [63].

Bonato et al. evaluated 111 chronic kidney disease patients, using 24-hour electrocardiogram, echocardiogram, and laboratory parameters [64]. Ventricular arrhythmia was found in 35% of the patients and was associated with age, increased* hemoglobin* level, and reduced ejection fraction [64].

A relationship was described between PTH level and sudden cardiac death, probably due to arrhythmia. The correlation between heart rate variability parameters and PTH serum level indicated the impaired autonomic function in patients with chronic renal failure [61]. Probably PTH has a role in the development of uremic cardiomyopathy, suggested by the correlations between PTH level and left ventricular hypertrophy in chronic renal failure [61].

Proteinuria is a marker of renal injury, detected earlier than the decline in glomerular filtration rate, and an independent risk factor for cardiovascular morbidity and mortality [65]. Microalbuminuria was related to prolonged QT interval, left ventricular hypertrophy, and ST-T changes in hypertensive patients, emphasizing the need of ECG monitoring and followup in patients with microalbuminuria [66]. High albumin excretion was related to left ventricular hypertrophy independent of age, blood pressure, diabetes, race, serum creatinine, or smoking, suggesting parallel cardiac damage and albuminuria [67]. Microalbuminuria was also independently associated with electrocardiographic markers of myocardial ischemia [68]. Urinary protein excretion reflects not only localized subclinical renal disease but also a generalized vascular endothelial dysfunction, and proteinuria was associated with inflammatory markers, including elevated C-reactive protein, fibrinogen, and asymmetric dimethylarginine (which causes endothelial dysfunction through inhibition of nitric oxide production), and also with circulating von Willebrand factor, soluble vascular cell adhesion molecule, and vascular endothelial growth factor [65]. Besides inflammation and endothelial dysfunction, thrombogenic factors may also link proteinuria and cardiovascular disease, including tissue plasminogen activator [65]. Proteinuria was also associated with insulin resistance and higher plasma insulin levels and altered lipid profile [69].

Iron overload in hemodialysis patients causes oxidative toxicity and may precipitate arrhythmias [70]. The high iron stores in patients with chronic ambulatory peritoneal dialysis patients were associated with higher QT dispersion [70]. Patients with QT dispersion longer than 65 ms had higher levels of serum ferritin and transferrin saturation than other patients undergoing chronic ambulatory peritoneal dialysis [70].

Patients with end stage renal disease have several factors which could predispose to the development of ventricular arrhythmia, including structural remodeling: myocardial fibrosis, left ventricular hypertrophy, the uremic cardiomyopathy, deposition of calcium, iron and aluminium within the heart tissue, endothelial dysfunction and vascular calcification; electrophysiological remodeling: slowing of conduction velocity, repolarization heterogeneities; pathophysiological triggers: ischemia, increased sympathetic activity, and inflammation; and dialytic triggers: electrolyte shifts, acid-base balance alterations, and hypotension [8, 42, 62, 64, 71]. The mentioned morphological changes could represent the substrate of the delayed and fractionated electrical conduction, enabling the appearance of late ventricular potentials [72]. Laboratory data, including serum electrolytes, especially potassium and calcium, pH, inflammatory markers, serum iron, proteinuria, hemoglobin, phosphates, and cystatin C, should be monitored, besides ECG QT intervals and T and R wave amplitude, in patients with end stage renal disease.

Cardioverter defibrillator implantation has been shown to reduce the risk of sudden cardiac death in patients with chronic kidney disease, in several randomized controlled trials. Many of those trials excluded patients on hemodialysis or with advanced chronic kidney disease [34]. The chronic kidney disease stage should be considered when an ICD should be implanted. On the other hand, chronic kidney disease modifies the efficacy of the ICD and sudden cardiac death is not necessarily a result of ventricular arrhythmia [34]. Hyperkalemia can augment T wave amplitude large enough to be detected by an ICD and deliver inappropriate ICD shocks [73]. Serum potassium should be monitored in ICD recipients with renal dysfunction and treated with angiotensin-converting enzyme inhibitors or angiotensin II receptor blockers, in order to prevent inappropriate ICD deliveries [73].

## 5. Hemodialysis

Cardiac disease and sudden cardiac death are increased in patients undergoing dialysis [74, 75]. Sudden cardiac death is responsible for about a third of total mortality among dialysis patients and is due to autonomic nervous system dysfunction and increased sympathetic activity, in particular [76]. Several electrocardiographic abnormalities were found in patients with chronic kidney disease undergoing a regular hemodialysis program, including QT interval prolongation and signs of left ventricular hypertrophy [77, 78].

Hemodialysis (HD) prolongs QTc in end stage renal disease patients, mainly related to rapid changes in electrolyte plasma concentrations. Important increases in QT interval and QT dispersion were found in both pre- and post-HD to levels only comparable to those recorded following myocardial infarction. However, the impact on QTc dispersion is less important in the absence of significant coexisting cardiac disease [77]. Patients on HD had longer QTd than patients on continuous ambulatory peritoneal dialysis, difference due to the higher* serum calcium* level [52]. QT and QTc dispersion were higher in patients who underwent hemodialysis, as well, in another study including 19 uremic patients. QTc dispersion positively and directly correlated with serum phosphates, and negatively to the calcium/phosphate ratio [79].

Patients, in whom a dialysis session determines an increase in QTc, started initially with significantly lower K and higher ionized calcium levels and displayed a greater reduction in calcium following dialysis [77]. Pre-HD plasma calcium appears to be the major determinant of QTc changes in HD patients. Thus, manipulation of plasma calcium through dialysate calcium may prove an effective mechanism to limit the arrhythmogenicity of a haemodialysis session, which may be important in dialysis subjects with known cardiac disease. Changes in ventricular repolarization duration associated with HD largely depend on the concentrations of calcium and potassium in the dialysis bath [80]. Similar results were obtained by Di Iorio et al., as the QT interval was significantly longer in patients with dialysate that contained the lowest concentrations of calcium and* potassium* and the highest concentration of* bicarbonate* [81].

The prevalence of late ventricular potentials in patients undergoing hemodialysis varied from study to study: 25% [72] versus 11% [52], and 7% continuous ambulatory peritoneal dialysis patients had positive late potentials. The differences were due to patient selection criteria and timing of SA-ECG after HD [52]. Signal averaged ECG parameters improved with HD due to fluid removal [82]. A prolongation of the SA-QRS duration after HD could be, probably, due to widening of the initial portion of the QRS, related to the acute reduction in serum potassium due to a generalized slowing of conduction in the myocardial fibers [72]. There was no relationship between SA-QRS duration prolongation and dialysis-induced changes in* serum sodium* and calcium or body weight changes [72]. LAS40 increased significantly postdialysis, correlated also with the changes of potassium [83].

Patients with end stage renal disease have tolerance for hyperkalemia, with less evident cardiac and neuromuscular consequences than in those with normal renal function [53]. No typical ECG changes of the T wave amplitude were found in HD patients with a high predialysis serum potassium concentration [53]. The tolerance to hyperkalemia is also explained by the slow rate of increase in serum potassium compared to the general population after excessive potassium ingestion [84].

Potassium level after HD is a very vulnerable point in arrhythmogenesis, considering that hypokalemia (<4 mEq/L), an insufficient decrease of potassium by hemodialysis or hyperkalemia (>5.6 mEq/L), are arrhythmogenic factors [83, 85, 86]. Hypokalemia contributes to reduced survival of cardiac patients and increased incidence of arrhythmic death [87]. Hypokalemia-induced arrhythmogenicity is due to slowed conduction, prolonged ventricular repolarization, and action potential duration associated with shortening of the effective refractory period enabling reentry, abnormal pacemaker activity, and early and delayed afterdepolarizations [87]. Checherita et al. evaluated the association of potassium level changes and arrhythmia in predialyzed and dialyzed patients and demonstrated that hypokalemia is a stronger risk factor than hyperkalemia for arrhythmia in chronic kidney disease patients [86].

As already mentioned, hypocalcemia prolongs the QT interval. QT dispersion increased with the use of low calcium-containing dialysate [88] and low calcium levels due to citrate anticoagulation are related to increased arrhythmic risk [89].

Using a low* magnesium* dialysate bath in 22 hemodynamically stable patients on maintenance hemodialysis without preexisting advanced cardiac disease did not significantly change QTc and QT dispersion [90].

Heart rate variability decreases in chronic HD patients, and the decrease is more important in diabetic uremic patients [19]. The changes of serum electrolytes and bicarbonate during HD did not affect heart rate variability [19]. On the other hand, depressed heart rate variability was associated with a higher risk of progression to end stage renal disease and suggested that autonomic dysfunction may lead to kidney damage [91].

Kyriakidis et al. concluded, in a study including 25 hemodialysis patients for chronic renal failure and undergoing Holter ECG monitoring for a continuous 48-hour period, that hemodialysis had no influence on type or frequency of arrhythmia, because they found only benign atrial arrhythmias and no complex ventricular arrhythmias [92]. The most important limitation of the mentioned study is the low number of patients.

Bignotto et al. found prolonged QT intervals in half of 179 patients on dialysis, a condition that was linked to left ventricular hypertrophy, presence of left bundle branch block, longer dialysis therapy period, older age, higher percentage of catheter use, and low body mass index [78].

Predialysis* hematocrit*,* oxygen content*,* serum urea*, and* osmolarity* were significantly different in chronic renal failure patients with and without arrhythmias and postdialysis serum phosphorus and osmolarity [93].

The adverse cardiomyopathic and vasculopathic milieu in chronic kidney disease favors the occurrence of supraventricular and ventricular arrhythmias, conduction abnormalities, and sudden cardiac death, exacerbated by electrolyte shifts, volume and acid-base balance shifts, blood pressure changes, diabetes mellitus and myocardial ischemia as comorbidities, sympathetic overactivity, inflammation, iron deposition and the deposition of calcium and aluminium salts in the heart tissue, impaired baroreflex sensitivity, and obstructive sleep apnea [72, 75, 94]. Changes of QT intervals during hemodialysis depend both on electrolyte and bicarbonate concentrations in the dialysate [81].

## 6. Kidney Transplantation

The risk of cardiovascular death is reduced in the renal transplant patients compared with those on dialysis, but still significantly greater than that of the general population [4]. Cardiovascular mortality in kidney transplant recipients is still high, especially in the first year of transplantation, and ventricular arrhythmia is one of the etiologies of sudden cardiac death [95]. Longer length on dialysis contributes to a greater prevalence of cardiovascular complications among kidney transplant recipients, especially from deceased donors [96]. The renal transplantation procedure may disturb the repolarization process, despite optimal hemodynamic or metabolic status [97].

Normalization of electrolytes and the acid-base status from a uremic state to the normal kidney function after successful kidney transplantation decreases the prolonged QT interval [98].

## 7. Conclusions

Ventricular arrhythmia and sudden cardiac death risk are increased in patients with renal failure, although not all studies have demonstrated it. Even mild reductions in kidney function can alter the electrophysiological properties of the myocardium and increase the risk of ventricular arrhythmias and sudden cardiac death.

The present review emphasizes important factors for the safety of patients with chronic kidney disease and enables multiple links between cardiology and nephrology departments and clinical laboratory, overcoming barriers, motivating nephrologists to consider cardiologists' opinion and laboratory data in order to prevent sudden cardiac death in end stage renal disease. All patients with kidney disease should be screened for cardiovascular disease.

Therapy of hypokalemia, hypocalcemia, and hypomagnesemia should be an emergency and performed simultaneously, under electrocardiographic monitoring in patients with renal failure, and sympathetic activity, serum phosphates and iron, PTH level, renal function, hemoglobin and hematocrit, pH, inflammatory markers, proteinuria and microalbuminuria, and osmolarity should be monitored, besides standard 12-lead ECG in order to prevent ventricular arrhythmia and sudden cardiac death. The relationship between new bystander biomarkers of renal function, including NGAL, and ventricular arrhythmia and sudden cardiac death should be assessed. Soluble ST2 and Galectin 3, biomarkers reflecting renal and cardiac fibrosis, could also be associated with an increased arrhythmia risk.

Electrocardiograms are low cost diagnostic tools for renal therapy centers, and nephrologists must consider QT interval, associated laboratory conditions, nutritional status, and QT interval prolonging drugs in their patients.
